# Suspected Lung Cancer with Suspicious Liver Lesions: Diagnostic Yield and Safety of Same-Day Bronchoscopy and Liver Biopsy in the Hands of a Pulmonologist

**DOI:** 10.3390/arm91010003

**Published:** 2023-01-18

**Authors:** Sina Ahmadzai, Jesper Koefod Petersen, Katrine Fjaellegaard, Paul Frost Clementsen, Uffe Bodtger

**Affiliations:** 1 Institute of Science and Environment, Roskilde University, 4000 Roskilde, Denmark; 2 Pulmonary Research Unit Zealand (PLUZ), Department of Respiratory Medicine, Zealand University Hospital, 4700 Næstved, Denmark; 3 Institute of Regional Health Research, University of Southern Denmark, 5230 Odense, Denmark; 4 Copenhagen Academy for Medical Education and Simulation (CAMES), 2100 Copenhagen, Denmark

**Keywords:** lung cancer, liver lesions, EBUS, same day procedure

## Abstract

**Highlights:**

**What are the main findings?**

- US-guided FNA from suspicious liver lesions are safe and feasible to perform in the same séance as bronchoscopy and EBUS in the hands of a pulmonologist.
- Same-day workup is a relatively new concept in lung cancer workup, with no prior investigation of the combination of endoscopy and liver biopsy.

**What is the implication of the main finding?**

- Same-day workup of lung tumors is efficacious, safe and timesaving.

**Abstract:**

Background: Bronchoscopy and endobronchial ultrasound (EBUS) are standard procedures for the diagnosis and staging of patients suspected of lung cancer. If the patient simultaneously presents with suspicious liver lesions, it is tradition to refer the patient to a radiologist for ultrasound-guided percutaneous liver biopsy. Objective: The aim of this study was to investigate the results and complications when the pulmonologist performs all three procedures in the same setting. Methods: We retrospectively identified patients who during 2018–2020 underwent invasive workup of suspected lung cancer and liver metastases with percutaneous liver lesion biopsy with or without same-day endoscopy (bronchoscopy and EBUS). We compared diagnostic yield and safety of liver lesion biopsy stratified by same-day endoscopy or not. Results: In total, 89 patients were included, of whom 28 patients (31%) underwent same-day endoscopy. All liver lesion biopsies were fine-needle aspiration biopsies performed by experienced pulmonologists. No complications were reported, and overall diagnostic yield was 88%. The diagnostic yield was significantly lower in the same-day endoscopy group (71% vs. 95%), and undergoing endoscopy was significantly associated with having fewer liver lesions, higher prevalence of lung cancer, and lower overall prevalence of a malignant diagnosis. Conclusion: Liver biopsy in the same session as endoscopy during lung cancer workup was feasible and safe. Confounding by indication was present in our study.

## 1. Introduction

Lung cancer incidence is increasing, and lung cancer is globally the most frequent cause of cancer death [1]. Despite improved access to advanced imaging such as computed tomography (CT) most patients are still diagnosed at an advanced stage, and around 40% have distant metastases at time of diagnosis [2]. Metastatic disease is associated with high symptom burden, re-admissions, and reduced survival [2,3,4], so minimizing delay in diagnosis and staging of suspected lung cancer is essential to commence optimal treatment to control disease and reduce symptoms [3].

The liver is a common site for lung cancer metastases, and both percutaneous and endoscopic routes allow cytopathological verification [4]. Imaging showing likely or evident liver metastases is highly suggestive for stage IV disease, and diagnosis and stage can be obtained by biopsy from any site. However, in cases of less unequivocal imaging results (possible or probable liver metastases), biopsies from several sites are warranted to provide a firm diagnosis and stage [5]. Traditionally, radiologists perform percutaneous biopsies from liver lesions and not pulmonologists, so patients in need of both procedures are usually called on separate days to pulmonologists for endoscopy and to radiologists for liver biopsy [6], with repeated registration, information and observation. 

Same-day workup is a new concept in lung cancer workup, defined as multiple invasive procedures in one day. Same-day workup of lung tumors—not reached by bronchoscopy or endobronchial ultrasonography (EBUS)—is efficacious, safe, and time-saving performed as either add-on percutaneous fine-needle aspiration (FNA) from peripheral lesions [7,8] or add-on endo-esophageal (EUS-B)-FNA from central lesion adjacent to the esophagus [9]. Additionally, ultrasound (US)-guided biopsies in the hands of pulmonologists from lung or pleural lesions is safe with a high diagnostic yield [10]. In the present study, we aim to investigate diagnostic yield and safety of same-day workup of suspected lung cancer with liver metastases in the hands of pulmonologists. We compare diagnostic yield and safety between patients undergoing US-guided FNA by pulmonologists from suspected liver metastases either alone or as add-on to bronchoscopy and EBUS. 

## 2. Methods

### 2.1. Design

We conducted a retrospective single-center study of patients undergoing liver biopsy for suspected malignancy at the Department of Respiratory Medicine, Zealand University Hospital Naestved between 1 January 2018 and 31 December 2020. All patients were identified using the ICD-10 procedure code for liver biopsy.

#### 2.1.1. Site

In Denmark, cancer referrals are organized in organ-specific cancer package pathways (CPP). 

Our unit is responsible for workup of suspected lung cancer and for suspected metastases (including in the liver) with unknown primary cancer, covering an area of around 400,000 citizens [8]. Invasive pulmonologists in our unit are familiar with sampling lesions in the liver using ultrasound-guided fine-needle aspiration (US-FNA) with a 22 G needle on a syringe. 

#### 2.1.2. Liver Biopsy

Liver biopsy was performed as an outpatient procedure with coagulation screen, hemoglobulin, and blood match analysis performed prior to biopsy. The liver was scanned using a LOGIQ S8 (GE Healthcare Wauwatosa, Wauwatosa, WI, USA) with the curvilinear C1-5 curved abdominal transducer (2–5 MHz), abdominal preset probe with the patient lying comfortably supported by cushions to maintain optimal body position. Skin at the planned biopsy site was cleaned with ethanol swabs, and skin and liver capsule were anaesthetized using 10 mL lidocaine-adrenalin, 20 mg/5 mg/mL under US-guidance. The skin was cleaned with chlorhexidine befusore three passes of a US-FNA with a 22G needle on a 10 mL syringe with direct observation of the needle in the lesion. Doppler was used if there was close proximity to vessels [4].

Vital signs were monitored before, immediately after, and every 15 min until 2 h after the end of the procedure. The patient was discharged thereafter for outpatient follow-up with biopsy results. Before leaving the department, the patient was carefully instructed in signs of bleeding and provided with written information on who to call.

All pulmonologists conducting endoscopy and liver biopsies had >5 years of experience with >500 procedures.

#### 2.1.3. Data Collection

All data were collected from electronic medical records and the Danish Pathology Registry linked to the Danish National Patient Registry, a national database containing results of all cyto- or histopathological analyses performed since 1990 in Denmark [11].

The medical files of patients with non-malignant liver biopsy results were revisited after 12 months to identify true- or false-negative cases. 

#### 2.1.4. Statistics

Statistical analyses were performed using RStudio Posit Software (2022.07.1+554, © 2009–2022 RStudio, PBC, Boston, MA, USA). Categorical data were described as number (*n*) and percentage (%), and continuous variables as median and interquartile range (IQR). Intergroup differences in categorical variables were analyzed with Chi2-test or Fisher’s exact test. Based on the number of true negatives (TN), true positives (TP), false negatives (FN), and false positives (FN), we calculated specificity, sensitivity, negative (NPV) and positive (PPV) predictive values, and diagnostic accuracy ((TP + TN)/ (TP + FP + TN + FN)) with corresponding 95% confidence intervals (CI) by 2 × 2 contingency tables. A *p*-value < 0.05 was considered statistically significant. 

## 3. Results

In total, 89 patients were included, 28 underwent same-day bronchoscopy, EBUS, and liver biopsy and 61 liver biopsy alone. In total, three pulmonologists performed all procedures. 

Baseline demographics, clinical data, and results of one day of invasive workup for the groups with and without same-day endoscopy are presented in Table 1. No adverse events needing intervention were recorded.

### 3.1. Final Diagnosis

Most patients (*n* = 83, 92%) had a final diagnosis of malignancy, predominantly lung cancer, see Table 2. Fewer cases of lung cancer were observed in the group undergoing liver biopsy only (*p* < 0.03), whereas this group included significantly more cases of gastrointestinal tract cancers, i.e., colorectal, upper GI, or primary liver cancer (36% vs. 16%, *p* < 0.05). No cases of malignancy were diagnosed in the follow-up period. 

### 3.2. Liver Biopsy Cytology

Of the 83 patients with a final diagnosis of malignancy, 78 patients (94%) had a liver biopsy with malignancy, see Table 1. One patient with known colorectal adenocarcinoma in the same-day endoscopy group had a false-positive liver biopsy showing malignant cells in a single liver lesion, but metastasis surgery revealed granulomatous inflammation only. Another patient in the same group had synchronous hepatocellular carcinoma and SCLC without liver metastases. None had a conclusive non-malignant liver biopsy result.

### 3.3. Diagnostic Values

The overall diagnostic yield of liver-FNA was 88% (78 conclusive/89 total liver biopsies), with significantly higher diagnostic yield in the liver biopsy only (58/61 vs. 20/28, *p* < 0.05; Fisher’s exact test). Table 3 depicts sensitivity, specificity, PPV, NPV, and diagnostic accuracy rates for each group and the total cohort. 

## 4. Discussion

This is the first study to explore ultrasound-guided liver biopsy in the hands of pulmonologists performed in the same séance as bronchoscopy and EBUS. We stratified patients according to completed same-day endoscopy or not. Everyday clinical practice implicates confounding by indication [12,13]. Patients with few liver lesions and reachable thoracic lesions need to undergo same-day endoscopy and liver biopsy, whereas patients with multiple liver lesions or no thoracic lesions do not [13]. In the first group, clinicians chose to refer patients to three procedures to ensure diagnosis and staging but estimated that only one procedure was needed in the latter group, who thus have a higher a priori likelihood of achieving a conclusive diagnosis. Consequently, a prospective randomized study would have been unethical. This is also reflected concerning diagnostic rates of US-guided FNA from liver lesions, which were significantly higher in the liver biopsy only group (Table 3).

However, our study suggests that US-guided FNA from liver lesions after same-day endoscopy is feasible and safe. 

We cannot compare our results with previous studies as none exist. A large retrospective study explored the outcome of radiologist-performed ultrasound-guided (n = 571) or CT-guided biopsy (n = 9) of possible liver metastases in 580 patients with known cancer and found a conclusive diagnosis was provided in >90% [14]. However, the true diagnostic accuracy is unknown, as the authors did not report data from 376 patients excluded with suspected liver metastases but no known malignancy. Another study reported that diagnostic accuracy can be increased to almost 100% by using intravenous contrast [15]. 

Our study has obvious strengths and limitations. Strengths include that no patients were lost to follow-up, and patients were identified using standardized procedure codes. All biopsies were conducted by experienced invasive pulmonologists, who perform these procedures on a daily/weekly basis. Limitations are mainly caused by the retrospective design [16]. We have no information on the prevalence of aborted procedures, as we were only able to identify patients by the liver biopsy procedure code. Our data support that group allocation was not random but based on clinical characteristics and the clinician’s choice of proceeding or not to endoscopy after liver biopsy, as the two groups differed significantly concerning liver lesion count (Table 1) and distribution of cancer diagnoses (Table 2), where lung cancer markedly dominated the same-day endoscopy group, and non-lung cancer dominated in the liver biopsy only group. Selection bias was present as we only report on patients with attempted liver biopsy. Thus, our cohort does not cover patients in whom a liver biopsy was planned but not performed due to localization, poor visualization, or other barriers. No selection was present concerning presumed difficulty of liver biopsy procedure, as we as a default perform all liver lesion biopsies in our clinic. Our data suggest that clinicians were highly able to allocate patients to endoscopy correctly, as the diagnostic yield in the “liver biopsy only” group was very high. Prospective studies are needed to provide information needed to report diagnostic values according to the Standards for Reporting Diagnostic Accuracy [17], and a randomized clinical trial is needed to clarify whether same-day endoscopy impairs diagnostic yield of liver biopsy when both endoscopy and liver biopsy are needed. 

## 5. Conclusions

This study suggests that US-guided FNA from suspicious liver lesions can be performed safely and conclusively the same day as bronchoscopy and EBUS with all procedures in the hands of the pulmonologist.

## Figures and Tables

**Table 1:** Demographic, basic clinical data, and results of one day of invasive workup in patients undergoing percutaneous fine-needle aspiration biopsy of liver lesions (*n* = 89).

	Same-Day Endoscopy *n* = 28	No Endoscopy *n* = 61	*p*-Value
Female	13 (46%)	31 (50%)	0.88
Age in years, median (IQR)	72 (66–76)	72 (65–78)	0.26
Earlier cancer diagnosis	7 (25%)	29 (47%)	0.07
Liver lesion			
Single	21 (75%)	11 (18%)	0.07
Multiple	7 (25%)	50 (82%)	
Liver lesion diameter, median (IQR) in mm.	23 (18–30)	26 (18–40)	0.41
Skin surface to liver lesion, median (IQR) in mm.	39 (31–48)	42 (29–56)	0.66
**Histopathology of liver biopsy**			
Malignancy in liver biopsy	21	58	0.34
Lung cancer	16 (75%)	27 (46%)	
Adenocarcinoma	4	13	
Squamous cell carcinoma	2	4	
Small-cell	10	8	
Other lung cancer	0	2	
Colorectal cancer *	2 (10%)	8 (14%)	
Upper Gastrointestinal cancer	1 (5%)	8 (14%)	
Primary liver cancer	1 (5%)	4 (7%)	
Breast Cancer	1 (5%)	5 (9%)	
Urogenital cancer	0	3 (5%)	
Other	0	3 (5%)	

* Including one case with known colorectal cancer and false-positive FNA with malignant cells: single-metastasis surgery with benign granulomatous inflammation.

**Table 2:** Final diagnoses stratified by same-day endoscopy or not.

	Same-Day Endoscopy *n* = 28	No Endoscopy *n* = 61
A. Subjects diagnosed with malignancy	24 * (86%)	59 (97%)
**Lung cancer**	**20 (83%)**	**27 (46%)**
Adenocarcinoma	7	13
Squamous cell carcinoma	2	4
Small-cell	11	8
Other lung cancer	0	2
**Other primary cancer**	**5 (20%)**	**32 (54%)**
Colorectal cancer	2 (8%)	9 (15%)
Upper Gastrointestinal cancer	1 (4%)	8 (13%)
Primary liver cancer	1 (4%)	4 (7%)
Breast Cancer	1 (4%)	5 (9%)
Urogenital cancer	0	3 (5%)
Other	0	3 (5%)
B. Subjects diagnosed with non-malignant diagnoses **	4 (14%)	2 (3%)

* *n* = 1: synchronous SCLC and primary liver cancer = 24 patients with 25 malignant diagnoses; ** *n* = 1: false-positive liver biopsy, see Table 1 and text.

**Table 3:** Diagnostic values of liver biopsies stratified by same-day endoscopy or not.

	TP	FP	FN	TN	Sensitivity (CI)	Specificity (CI)	PPV (CI)	NPV (CI)	Diagnostic Accuracy	Diagnostic Yield
**Same-day** **endoscopy**	20	1	4	3	83% (67–96%)	75% (19–99%)	95% (76–100%)	43% (10–82%)	82% (63–94%)	71% (51–87%)
**No** **endoscopy**	58	0	1	2	98% (91–100%)	100% (16–100%)	100% (94–100%)	67% (9–99%)	98% (91–100%)	95% (86–99%)
**All liver** **biopsies**	78	1	5	5	95% (88–99%)	83% (36–100%)	99% (93–100%)	56% (21–96%)	94% (87–98%)	88% (79–94%)

TP—true positive; FP—false positive; FN—false negative; TN—true negative; PPV—positive predictive value; NPV—negative predictive value; CI—confidence Interval.

## Data Availability

The data presented can be obtained by written contact to the corresponding author.
